# Health literacy in patients with pulmonary embolism: development and validation of the HeLP (Health Literacy in Pulmonary Embolism)-Questionnaire

**DOI:** 10.3389/fpubh.2023.1167499

**Published:** 2023-08-29

**Authors:** Simone Fischer, Anja Kalch, Constanze Küchler, Aliscia Rebecca Albani, Helena Bilandzic, Dirk Horenkamp-Sonntag, Thomas M. Berghaus, Christine Meisinger, Inge Kirchberger

**Affiliations:** ^1^Epidemiology, Faculty of Medicine, University of Augsburg, Augsburg, Germany; ^2^Institute for Medical Information Processing, Biometry and Epidemiology (IBE), LMU München, Munich, Germany; ^3^Department of Media, Knowledge and Communication, University of Augsburg, Augsburg, Germany; ^4^Techniker Krankenkasse, Healthcare Management, Hamburg, Germany; ^5^Department of Cardiology, Respiratory Medicine and Intensive Care, University Hospital Augsburg, Augsburg, Germany

**Keywords:** health literacy, pulmonary embolism, disease-specific questionnaire, psychometric evaluation, factor analyses, item response theory

## Abstract

**Background:**

Pulmonary embolism (PE) is a common cardiovascular disease and health literacy is necessary to deal with its consequences after the acute event. The aim of this study was to develop and validate a new questionnaire to measure PE-specific health literacy.

**Methods:**

A mixed-methods design with qualitative and quantitative elements was used in the development process. A literature review about health literacy concepts and instruments and interviews with patients with PE and clinicians were conducted. Quantitative analyses included factor analyses, item response theory with a graded partial credit model, and reliability analyses in different test and validation samples. Furthermore, convergent and known-groups validity and responsiveness were assessed.

**Results:**

The qualitative results supported a concept of PE-related health literacy with four main topics: dealing with PE-related health information, disease management, health-related selfcare, and social support. An initial item pool of 91 items was developed. Further interviews and an online survey with patients with PE (*n* = 1,013) were used to reduce the number of items and to confirm structural validity. Confirmatory factor analyses in the final evaluation study with patients with PE (n = 238) indicated a good model fit of the four-factor structure. The Health Literacy in Pulmonary Embolism (HeLP)-Questionnaire showed good reliability (Cronbach’s alpha: 0.82 to 0.90). All four subscales were responsive toward receiving a brochure with PE-related health information.

**Conclusion:**

The newly developed German HeLP Questionnaire comprises 23 items in four domains and showed good psychometric properties. Further evaluation of the questionnaire in different samples of patients with PE is needed.

## 1. Introduction

Pulmonary embolism (PE) is globally the third most common cardiovascular disease, after myocardial infarction and stroke, and the incidence rates are rising (1). PE is potentially fatal and patients surviving a PE may suffer from long-term consequences including persistent dyspnoea, impaired physical functioning, right heart failure, and chronic thromboembolic pulmonary hypertension (2). Bleeding risk due to anticoagulant medication intake and fears regarding a potential recurrent event may also affect mental health (3). Studies revealed that a considerable amount of patients suffer from symptoms of depression or anxiety after PE (4–6). The acute event is usually followed by an outpatient treatment with anticoagulant medication for at least 3 to 6 months or even longer. Patients are faced with finding a way to cope with their disease, adhering to the treatment, recovering, and preventing further events. For these processes patients’ health literacy plays an important role in the active and responsible management of their disease. Health literacy comprises the skills to manage a disease and promote one’s own health. Among many existing definitions of health literacy, a comprehensive definition was developed in the European Health Literacy Survey (HLS-EU) and has meanwhile been spread widely: “Health literacy is linked to literacy and entails people’s knowledge, motivation and competences to access, understand, appraise and apply health information in order to make judgments and take decisions in everyday life concerning health care, disease prevention and health promotion to maintain or improve quality of life during the life course” (7). The World Health Organization identified health literacy as a key determinant of health. Limited health literacy results in a variety of negative health related outcomes, such as “less healthy choices, riskier behavior, poorer health, less self-management and more hospitalization” (8). Among older patients, poor health literacy is associated with higher mortality rates (9). In a study about patients with chronic obstructive pulmonary disease (COPD) poor health literacy was associated with greater disease severity, greater helplessness, worse respiratory-specific quality of life, and more frequent utilization of COPD-related emergency health-care (10). Since health literacy has been shown to have a crucial impact on several general and disease-specific health outcomes, sound health literacy measures are needed to identify potential deficits. A number of instruments for measuring general health literacy already exist. Some focus on functional health literacy and are linked to reading and numerical literacy. Others, e.g., the HLS-EU Questionnaire (11) or the Health Literacy Questionnaire (HLQ) (12), involve a more comprehensive definition of health literacy and comprise dimensions about engagement with health care providers, the appraisal of health information or the existence of sufficient social support. Health literacy is important to promote general health, but different diseases may require different competencies. For some diseases, such as diabetes, multiple sclerosis, or heart failure, specific health literacy tools have already been developed (13–16). Similarly, in addition to generic health literacy (e.g., understanding information about the disease), patients with PE also face specific challenges (e.g., dealing with anticoagulant medications) that need to be mastered. To our knowledge, no instrument for measuring PE-related health literacy exists. The aim of this study was to develop a questionnaire that addresses PE-specific issues of health literacy and to evaluate its psychometric properties.

## 2. Methods

### 2.1. Study aim and design

**Figure 1:**
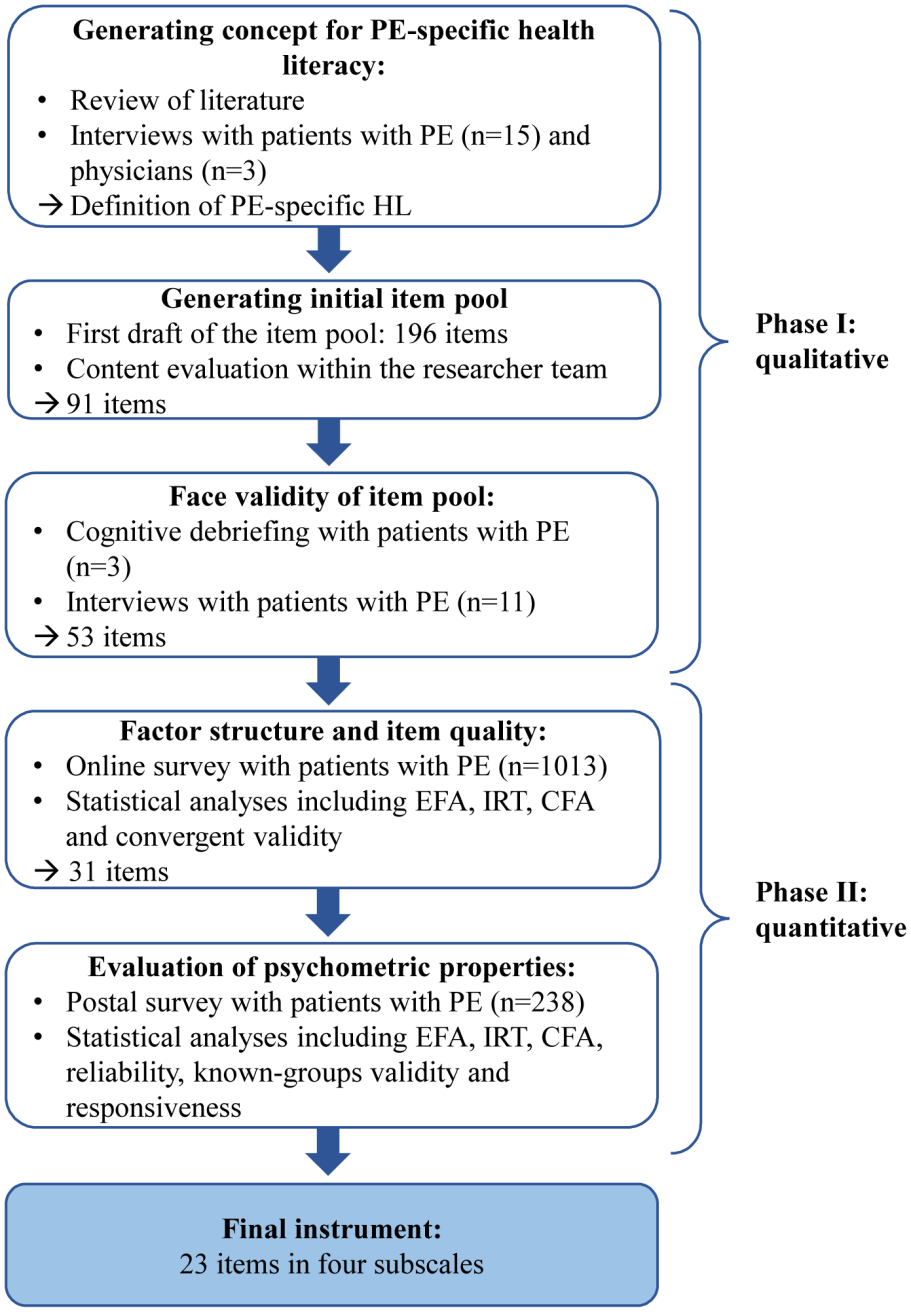
Development and validation process of the HeLP questionnaire.

The Health Literacy in Pulmonary Embolism (HeLP)-Questionnaire was developed and validated between May 2020 and November 2022 as a part of the research project INFO-LE (Evidence-based health information for patients with PE) which is a collaboration between epidemiologists and communication scientists of the Ludwig-Maximilian-University Munich and the University of Augsburg. The main goal of the project was to develop evidence-based health information for patients with PE which was accompanied by the development of a PE-specific health literacy questionnaire. For the development and validation process, we used a mixed-methods design with qualitative and quantitative approaches (Figure 1). We planned all steps and analyses in accordance with the Consensus Based Standards for the Selection of Health Measurement Instruments (COSMIN) checklist to assure high methodological quality (17). The study was approved by the Ethics Committee of the Ludwig-Maximilians-Universität München (Date of approval: 6 July 2020. Reference number: 20–452). The study was performed according to the Declaration of Helsinki.

### 2.2. Phase I: literature search and qualitative interviews

The development process started with a literature review about concepts and measures of health literacy. To develop a comprehensive concept of PE-specific health literacy we explored experiences of patients and physicians by conducting interviews. Patients were recruited from the LEA cohort study and via Social Media channels. The LEA study is a long-term observational cohort study including patients 18 years and older with PE who were treated at the University Hospital Augsburg. Patients with incident or recurrent confirmed PE diagnosis based on multidetector CT pulmonary angiography or ventilation–perfusion lung scanning are included in the cohort (18). For our interviews, we tried to include a heterogenous sample of patients with PE to cover different perspectives. Patients received postal information about the study and an invitation for a face-to-face interview. Due to restrictions regarding the COVID-19 pandemic, some interviews were carried out by phone or via an online video call. The topics comprised the definition of health literacy, specific PE-related aspects, and how high and low health literacy related to PE could be defined. The interviews were analyzed using qualitative content analysis. As a result, a concept of PE-specific health literacy and a list of questionnaire items were developed. A second round of patient interviews including cognitive debriefing was carried out to investigate the face validity of the first item pool and to eliminate repetitive or inadequate items (19).

### 2.3. Phase II: quantitative analyses of online and postal survey

The first part of the quantitative phase consisted of an online survey of members of a German health insurance company, who were at least 18 years old, and had at least one PE event in the past 2 years. The patients were contacted by the health insurance company and received an access link to the online questionnaire. The survey data were used to investigate the factorial structure using explanatory factor analysis (EFA) and confirmatory factor analysis (CFA). The quality of each item was additionally investigated using item response theory (IRT). In this step, the sample was divided into a test and a validation sample. The test sample was used for EFA and IRT. In the EFA, we used weighted least squares (WLS) estimation and different rotation methods to decide which fits the theoretical concept best (20). Bartlett’s Test of Sphericity and Kaiser–Meyer–Olkin (KMO) index were used to determine the appropriateness of the sample for factor analysis (21). Parallel analyses and Empirical Kaiser Criterion (EKC) were used to examine the number of factors to be extracted. Items with factor loadings < 0.3 or cross loadings > 0.3 and with a difference between loadings < 0.2 were excluded. EFA was repeated with the remaining items until an interpretable solution was obtained. After EFA, the items were analyzed using IRT. Since unidimensionality is an important assumption of IRT, a graded partial credit model (GPCM) was calculated separately for each previously extracted factor. We inspected slope (a) parameters for discrimination and location (b_i_) parameters for item difficulty, item trace lines and test information curves. Items with unordered b_i_ parameters or a < 1 were eliminated unless they were seen as indispensable for the measured construct.

After this item reduction process, a second EFA was conducted to identify possible changes to the first factor solution. In the next step, a CFA using data of the validation sample was conducted. For assessing model fit we used different global goodness of fit indices: Chi-square test statistics (χ^2^), Tucker-Lewis Index (TLI), Comparative Fit Index (CFI), Root Mean Square Error of Approximation (RMSEA) and Standardized Root Mean Square Residual (SRMR). For good model fit, the chi-square test statistics should be non-significant and the ratio χ^2^/df < 2 (or at least < 3), TLI and CFI ≥ 0.95 (or at least ≥ 0.90) (22), RMSEA ≤ 0.05 (or at least ≤0.08) and SRMR ≤0.05 (or at least ≤ 0.10) (23). For handling not normally distributed data, we used robust maximum likelihood (MLR) and full information maximum likelihood method to account for missing data. Modification indices were used to identify local dependency which was modeled where necessary. Spearman correlation coefficients with the validated German version of the Health Literacy Questionnaire (HLQ) were calculated. The HLQ is an instrument that measures generic health literacy and comprises nine different domains: “feeling understood and supported by healthcare providers,” “having sufficient information to manage my health,” “actively managing my health,” “social support for health,” “appraisal of health information,” “ability to actively engage with healthcare providers,” “navigating the healthcare system,” “ability to find good health information,” and “understanding health information well enough to know what to do” (12). For convergent validity, we assumed at least moderate correlations with some of the HLQ scales.

The last part was an evaluation study based on a postal survey of patients with PE who were treated at the University Hospital Augsburg. We examined completeness of the data and floor and ceiling effects which are presented as proportions of participants with minimal and maximal possible scores. We considered them acceptable if they accounted for <15% (24). Another EFA and CFA was conducted to confirm the factor structure in this sample. We tested psychometric properties including internal consistency, known-groups validity and responsiveness of the HeLP questionnaire with all finally selected items. Internal consistency was measured by Cronbach’s alpha, McDonald’s omega, and average inter-item correlation. Cronbach’s alpha and McDonald’s omega coefficients were considered appropriate if they ranged between 0.70 and 0.95 (24). Known-groups validity requires the questionnaire to discriminate between groups that are known to differ on the construct of interest (25) and was tested with Mann–Whitney *U*-tests and rank-biserial correlation as effect size (r < 0.3: small, r = 0.3–0.5: moderate, r > 0.5: large) (26). The variables education, job situation and age were selected for building the groups.

Some of the participants completed the questionnaire twice to test responsiveness which was considered as the ability of the measure to detect change. After the first assessment, they received a brochure with evidence-based health information about PE which was newly developed as a part of the INFO-LE study. We hypothesized that PE-related health literacy may be improved by this intervention and conducted Wilcoxon signed-rank tests with rank-biserial correlation (r) as effect size for all four subscales before and after the participants have received the brochure. Additionally, we report the standardized response mean (SRM) as a second effect size which is calculated by dividing the mean change between the measurements by the standard deviation of the change score (27). All analyses were performed using the statistic software R version 4.2.1 (28) mainly with the packages “lavaan,” “mirt,” and “psych.”

## 3. Results

### 3.1. Phase I

#### 3.1.1. Developing a concept of PE-specific health literacy and an initial item pool

On the basis of a comprehensive literature review about general and disease-specific health literacy and existing instruments, three interviews with physicians (pulmonologists and cardiologists) and 15 interviews with patients with PE, a concept of *PE-specific health literacy* was developed. In accordance with Begoray and Khan (29) and Sorensen (7) we defined the concept as follows:

“*Pulmonary embolism (PE)-specific health literacy covers people’s motivation, knowledge and competences to access, understand, appraise, and apply information on PE to engage with the demands of PE health care, prevention and health promotion to maintain and promote post PE health and health-related quality of life.”*

The interviews revealed that four topics related to health literacy seem to be important for patients with PE, namely: dealing with PE-related health information, actively managing the disease, selfcare, and seeking and accepting social support. An item pool with initially 196 items was created, covering these four domains. A 5-point Likert scale was chosen as response format ranging from “very difficult,” “rather difficult,” “little difficult,” “rather easy,” to “very easy.” After repeated evaluation in terms of item wording and content within the project team, we rephrased or eliminated several items, leaving 91 items to be tested with patients for the first time.

#### 3.1.2. Face validity of first item pool

The first three interviews included cognitive debriefing. In this process, detailed questions about the meaning, relevance and difficulty of each item as well as the appropriateness of the response categories were examined. The following 11 interviews included more general questions about the item pool. All participants of the interviews completed the entire pool of 91 items. Six patients were involved in more than one interview, e.g., shared their experience with PE in the first round and were also part of the cognitive debriefing of the first item pool. Sample characteristics of all 29 interviewed participants are shown in Supplementary Table S1. Participants were 19 to 79 years old, 14 were male, 22 of them were still taking anticoagulant medication, and the time since their last PE event varied from 1 month to 8 years. The direct feedback from the target group provided us with valuable insights into the appropriateness of the items. Misleading or repetitive items were eliminated and we were able to shorten the questionnaire to 53 items which were then tested in the quantitative analyses.

### 3.2. Phase II

#### 3.2.1. Factor structure and item quality

For the online survey, 3,200 patients were contacted and 1,154 participated, of whom 1,118 reported that they actually had at least one PE. In this step, we examined the distribution and frequencies of the response categories. One response category named ‘not applicable for me’ was included at this stage to identify items that do not apply to a large proportion of patients with PE. However, none of the items was excluded for this reason. For further analyses, we excluded cases who did not complete all questions about PE-specific health literacy. Two more participants were excluded because they solely used the category ‘not applicable for me’. The final sample included 1,013 participants and was randomly divided in a test sample (*n* = 505) and a validation sample (*n* = 508). Sample characteristics of the online survey are shown in Supplementary Table S2. With 29% most of the patients were in the age group between 61 to 70 years. Thirty-four percent of the patients were female and about 18% had two or more PE events. The median of the time interval since their last PE was 18 months.

Based on the investigation of item distribution, three items were excluded due to very high ceiling effects of 40%, 31%, and 60%, respectively. One item about medication intake showed a similar high ceiling effect (54%), but remained in the item pool because of its relevance in terms of content. We examined the correlation matrix and excluded five items with very high inter-item correlation (r = 0.79 to r = 0.87). We then conducted an EFA with the test sample to examine the underlying factor structure of our item pool. Bartlett’s test and KMO confirmed the adequacy of our sample (KMO = 0.95 and χ^2^ = 14920.55, *p* < 0.001). Both, EKC and parallel analysis, suggested four factors to be extracted from the data. We also tried three and five factor solutions and different oblique rotation methods, but four factors with bentler rotation resulted in the best interpretable solution. During EFA we removed eight items that did not load on any factor above 0.3 or showed cross loadings on two or more factors.

We conducted EFA before using IRT models to assure that the assumption of unidimensionality is met. Therefore, one IRT model (GPCM) was fitted separately for each factor. Six items with disordered thresholds or very low slopes were excluded. Four items remained in the questionnaire despite low slopes or slightly disordered thresholds because of their theoretical relevance. They addressed medication intake and dealing with symptoms of PE such as dyspnoea, which were considered important by the interviewees. The test information of all factors showed a peak in the area slightly below average.

After reducing the number of items, we conducted EFA again to confirm that the factor solution has not changed. The factor loadings at this stage ranged from 0.39 to 0.88 in factor 1, from 0.32 to 0.90 in factor 2, from 0.32 to 0.89 in factor 3 and from 0.53 to 0.84 in factor 4. Correlation between the four factors ranged from 0.45 to 0.66 and cumulative variance explained by the four factors was 54%.

To confirm this four-factor structure in the validation sample we conducted a CFA. After inspection of modification indices, we correlated four error terms that were supported by theoretical rationale and each within one factor. Two questions cover finding information, two address understanding information, two relate to checking the quality and sources of information, and two were about following physicians’ recommendations. Therefore, they all had a high overlap in content. After this step, the model yielded acceptable fit statistics: χ^2^ = 1028.371, df = 424, *p* < 0.001, χ^2^/df = 2.4, TLI = 0.896, CFI = 0.905, RMSEA = 0.058 (0.054; 0.063), SRMR = 0.066.

We built mean scores for each subscale with higher scores indicating better PE-specific health literacy. To examine convergent validity, we correlated our new factor scores with the scales of the HLQ. All correlation coefficients were statistically significant (*p* < 0.05) and are shown in Table 1. Highest correlation coefficients for each factor varied between 0.50 for factor 2 and 0.69 for factor 4.

**Table 1:** Convergent validity with the HLQ.

HLQ scales	F1	F2	F3	F4
Feeling understood and supported by healthcare providers	0.28	0.31	0.28	0.45
Having sufficient information to manage my health	0.51	0.40	0.44	0.52
Actively managing my health	0.16	0.27	0.35	0.21
Social support for health	0.27	0.34	0.37	0.45
Appraisal of health information	0.29	0.08	0.10	0.20
Ability to actively engage with healthcare providers	0.55	0.50	0.49	0.67
Navigating the healthcare system	0.56	0.50	0.56	0.69
Ability to find good health information	0.68	0.38	0.37	0.50
Understand health information well enough to know what to do	0.53	0.39	0.32	0.34

Spearman correlation coefficients; all statistically significant with *p* < 0.05. F1, Dealing with PE-related health information; F2, Disease management; F3, Health-related selfcare; F4, Social support.

The analysis of the online survey resulted in a pre-final version of 31 items in four subscales.

#### 3.2.2. Evaluation study with final psychometric properties

For the evaluation study, we recruited 300 patients who received an information brochure and the pre-final version of the health literacy questionnaire. Finally, the response rate was 80% with 240 participants. Some of them were asked to complete the questionnaire twice, before and after receiving the brochure. The mean time interval between the first and the second questionnaire after the brochure was 32 ± 14 days. Since two of them did not return the second questionnaire, our final sample included 238 patients. Patients’ characteristics are presented in Table 2. Forty-five percent were women and the age ranged from 21 to 91 years with a mean age of 63 ± 15 years. Seventeen percent had already two or more PE events. The mean time between the PE event and study participation was 32 ± 21 months.

**Table 2:** Sample characteristics of the evaluation study.

Variable	*N*	
**Mean (SD)**		
Age	238	63.2 (14.6)
Time since last PE event (months)	220	32.1 (21.1)
***n* (%)**		
Sex	238	
Men		129 (54.2)
Women		109 (45.8)
Marital status	237	
Married		151 (63.7)
Single		35 (14.8)
Divorced		25 (10.5)
Widowed		26 (11.0)
Living alone	236	
Yes		66 (28.0)
No		170 (72.0)
Education level	237	
≤9 years school education		84 (35.4)
10 years school education		56 (23.6)
≥12 years school education		32 (13.5)
University degree		65 (27.4)
Occupation	235	
Employed (part or full time)		90 (38.3)
Not employed		13 (5.5)
Retirement		130 (55.3)
In education		2 (0.9)
Number of PE events	234	
1		194 (82.9)
>1		40 (17.1)
Prior diseases		
Thrombophila	223	67 (30.0)
Diabetes	221	23 (10.4)
Hypertension	226	108 (47.8)
Chronic heart failure	216	25 (11.6)
Myocardial infarction	218	11 (5.0)
Stroke	219	11 (5.0)
Mental disorder	220	25 (11.4)
Pulmonary hypertension	217	6 (2.8)
Cancer	224	43 (19.2)

PE, pulmonary embolism.

For all items, missing values were below 5%, indicating good acceptability. Two items were eliminated due to high mean inter-item correlation > 0.75 and redundancy of content. Items related to medication intake (final items 9 and 10) showed ceiling effects but again remained in the questionnaire because of their theoretical relevance. Another exploratory factor analyses (following the same procedure as for the data of the online survey) resulted in the deletion of three items with insufficient factor loadings. GPCMs were again fitted separately for each factor to identify unordered thresholds. The final questionnaire included only three items without ordered thresholds (Table 3). Thresholds ranged between −2.77 and 2.28 for “dealing with health information,” −2.74 and 1.07 for “disease management,” −2.91 and 1.71 for “health-related selfcare,” and between −1.89 and 1.43 for “social support” (Supplementary Table S3). Results of the CFA with MLR estimation approved a four-factor model. The inspection of modification indices led to the exclusion of three items due to high correlations and cross loadings and an inclusion of covaried error-terms between the items regarding medication intake (item 9 and item 10). The final model yielded good fit statistics: χ^2^ = 381.353, df = 223, *p* < 0.001, χ^2^/df = 1.7, CFI = 0.931, TLI = 0.921, RMSEA = 0.060 (0.050, 0.070) and SRMR = 0.061. All factor loadings were significant and above 0.5 (Table 3; Supplementary Figure S1). The correlations between the four latent factors ranged from 0.33 to 0.66. Cronbach’s alpha and McDonald’s omega indicated very good reliability ranging from 0.82 to 0.90 and 0.78 to 0.91, respectively. Average inter-item correlation ranged between 0.49 and 0.61. The final subscales showed no relevant floor or ceiling effects (Table 4).

**Table 3:** Item content and properties.

		Mean	SD	Ordered thresholds (GPCM)	Factor loadings (CFA)	R^2^
**Dealing with health information**						
Item 1	Find information about pulmonary embolism that I can understand well.	3.35	0.97	Yes	0.67	0.45
Item 2	Understand advice or instructions from physicians and therapists.	3.86	0.84	Yes	0.75	0.56
Item 3	Understand written information about pulmonary embolism.	3.69	0.88	Yes	0.78	0.60
Item 4	Understand how I can reduce the risk of pulmonary embolism.	3.89	0.90	Yes	0.77	0.59
Item 5	Evaluate the quality of information about pulmonary embolism.	3.21	1.03	Yes	0.67	0.45
Item 6	Make decisions for my health after pulmonary embolism using health information.	3.49	0.89	Yes	0.73	0.53
Item 7	Recognize symptoms of an occurring pulmonary embolism.	3.22	0.98	Yes	0.54	0.29
**Disease management**						
Item 8	Follow recommendations on how to behave in order to avoid another pulmonary embolism (e.g., wear compression stockings, stop smoking, etc.)	3.86	0.96	Yes	0.72	0.52
Item 9	Take the prescribed medication (anticoagulants) regularly and on time.	4.48	0.81	No	0.50	0.25
Item 10	Deal well with the side effects of the medication (anticoagulants).	4.17	0.87	No	0.57	0.33
Item 11	Actively reduce my risk of thrombosis.	3.74	0.87	Yes	0.80	0.65
Item 12	Follow the advice of my physicians and therapists.	4.08	0.81	Yes	0.76	0.58
**Health-related selfcare**						
Item 13	Find the right balance of activity and rest.	3.62	0.88	Yes	0.75	0.56
Item 14	Take care of what is good for my health.	3.78	0.83	Yes	0.83	0.69
Item 15	Let my body rest when it is necessary.	3.83	0.94	Yes	0.84	0.71
Item 16	Take care of myself and my health needs on a regular basis.	3.63	0.90	Yes	0.86	0.75
Item 17	Recognize changes in my physical well-being.	3.70	0.92	Yes	0.73	0.54
Item 18	Set boundaries to not overload myself.	3.42	0.95	Yes	0.68	0.46
**Social support**						
Item 19	Find the right health care provider for my needs.	3.32	1.10	Yes	0.74	0.55
Item 20	Call upon someone when I have anxiety or depression about the pulmonary embolism.	3.30	1.18	Yes	0.69	0.47
Item 21	Ask my physicians and therapists if I am unsure about anything.	3.87	0.95	No	0.67	0.45
Item 22	Talk to other people about pulmonary embolism.	3.20	1.15	Yes	0.69	0.48
Item 23	Seek support from others when I need help dealing with pulmonary embolism.	3.27	1.11	Yes	0.84	0.70

CFA Model fit: χ^2^/df = 1.7, robust CFI: 0.93, robust TLI: 0.92, robust RMSEA and CI: 0.060 (0.050, 0.070), SRMR: 0.061.

**Table 4:** Acceptability and internal consistency.

Scale	Number of items	Missings % (*n*)	Floor effects %	Ceiling effects %	Cronbach’s alpha	McDonald’s omega	Average inter-item correlation
Dealing with health information (HI)	7	1.3 (3)	0	1.3	0.87	0.87	0.49
Disease management (DM)	5	0.4 (1)	0	11.3	0.82	0.78	0.49
Health-related selfcare (SC)	6	0 (0)	0	2.9	0.90	0.91	0.61
Social support (SU)	5	2.5 (6)	0	6.7	0.85	0.85	0.52

**Figure 2:**
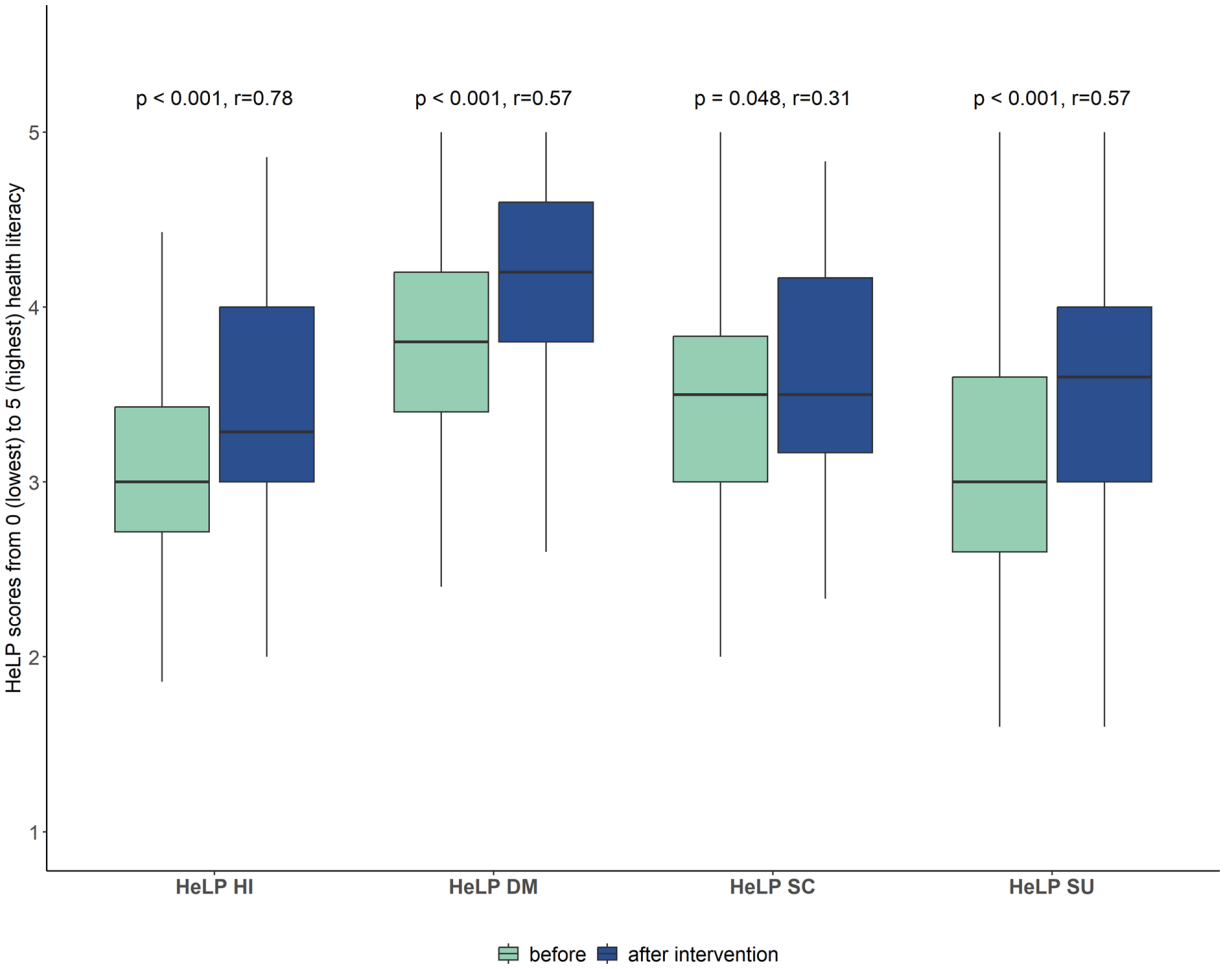
Responsiveness of the HeLP-questionnaire. Scores on the four subscales before and after receiving the brochure with evidence-based health information (Wilcoxon signed rank test, *n* = 58). Higher scores indicate higher health literacy.

Known-groups-validity was tested for age, years of education and job situation. Younger patients and patients with a higher education level showed significantly higher scores on the scale for “dealing with health information.” Scores on “health-related selfcare” were higher for older patients, patients who are retired or not employed, and patients who live alone. Effect sizes were small, ranging from 0.16 to 0.27 (Table 5). After receiving the brochure with PE-related health information, patients showed significantly higher scores on all four subscales with moderate (r = 0.31) to large (r = 0.78) effect sizes (Figure 2), indicating good responsiveness of the questionnaire. The calculation of the SRM revealed small to moderate effects with SRM = 0.54 for the scale “dealing with health information,” SRM = 0.30 for “disease management,” SRM = 0.20 for “health-related selfcare,” and SRM = 0.31 for “social support.”

**Table 5:** Known-groups validity.

	Groups		*p*-value^b^	|r|^c^
**Age**	**<65 years**	**≥65 years**		
GI	3.6 (3.3, 4.1)^a^	3.3 (3.0, 3.7)	0.013^*^	0.19
KM	4.0 (3.6, 4.4)	4.2 (3.8, 4.6)	0.009^*^	0.19
SF	3.5 (3.0, 4.0)	3.8 (3.3, 4.3)	<0.001^*^	0.27
SU	3.4 (2.8, 4.0)	3.4 (3.0, 4.0)	0.627	-
**Education**	**<10 years**	**≥10 years**		
GI	3.3 (3.0, 3.9)	3.6 (3.1, 4.1)	0.028^*^	0.17
KM	4.2 (3.6, 4.7)	4.1 (3.6, 4.4)	0.123	-
SF	3.8 (3.2, 4.3)	3.7 (3.2, 4.0)	0.046^*^	0.16
SU	3.6 (3.0, 4.0)	3.4 (3.0, 4.0)	0.493	-
**Job situation**	**Retired/not employed**	**Employed/in education**		
GI	3.3 (3.0, 3.9)	3.7 (3.3, 4.1)	<0.001^*^	0.26
KM	4.2 (3.6, 4.6)	4.0 (3.6, 4.4)	0.178	-
SF	3.8 (3.2, 4.3)	3.5 (3.0, 3.9)	0.001^*^	0.25
SU	3.4 (2.8, 4.0)	3.6 (3.0, 4.0)	0.399	-
**Living situation**	**Living alone**	**Living with other persons**		
GI	3.4 (3.0, 4.0)	3.5 (3.1, 4.0)	0.486	-
KM	4.1 (3.6, 4.6)	4.2 (3.6, 4.6)	0.855	-
SF	3.8 (3.2, 4.5)	3.7 (3.2, 4.0)	0.025^*^	0.19
SU	3.4 (2.7, 4.1)	3.4 (3.0, 4.0)	0.818	-

a Median (Q_25,_ Q_75_).

b Mann–Whitney *U*-test.

c Rank-biserial correlation.

* Significant with alpha = 0.05.

## 4. Discussion

In this study, we developed the first questionnaire about PE-related health literacy using a mixed-methods approach. A literature review on concepts and measures of health literacy as well as 29 interviews with patients and three interviews with physicians were conducted and qualitatively analyzed. Quantitative analyses included factor analyses, methods of IRT, reliability analyses, convergent validity, known-groups validity and responsiveness. Data basis was an online survey with 1,013 participants and a postal survey with 238 patients with PE. We found four domains that are relevant for PE-related health literacy: “dealing with health information,” “disease management,” “health-related selfcare,” and “social support.” The scale “dealing with health information” comprises items about accessing, understanding, appraising, and applying health information. These four competencies are in accordance with the definition of health literacy by Sørensen et al. (7) and are also captured in the related questionnaire HLS-EU-Q47 (11). The items of the scale ‘disease management’ ask whether patients have difficulty regarding PE-specific recommendations such as reducing their thrombosis risk or medication intake. The scale “health-related selfcare” contains aspects about physical and mental well-being including resting and setting boundaries in favor of their own health. The fourth scale about “social support” includes items about seeking support from various contact persons if help or advice in dealing with the PE is needed. Many health literacy questionnaires focus on health information. Disease-specific questionnaires can additionally address competencies that are directly related to the reality of patients’ disease experiences. We included the scale “disease management” to reveal specific problems with handling the disease and its consequences such as medication intake and other thrombosis prophylaxes. Items regarding disease management are also part of health literacy questionnaires for other diseases such as multiple sclerosis (13). Since challenges with setting boundaries and dealing with limited physical performance were frequently mentioned in the interviews with patients, it seemed important to additionally include items about “health-related selfcare.” This demonstrates the benefits of developing a disease-specific instrument and of the involvement of patients in the development process in order to consider PE-specific challenges reported by the patients.

Overall, the final questionnaire obtained good psychometric properties. A four-factor structure was confirmed by CFA with adequate fit statistics. The final model showed significant chi-square test statistics with *p* < 0.001, which would indicate bad fit, but the ratio χ^2^/df was 1.7, which is considered as good fit. It has previously been discussed that robust estimation can lead to over-rejection by corrected chi-square test statistics (30). Covaried error-terms between the two items about medication intake helped to improve the model fit but the respecification is supported by theoretical rationale and within the same scale. No overall score was built because the four subscales are considered to be interpreted separately. The four domains represent different aspects and we assume that an overall score would mask patients’ individual needs in specific areas of health literacy. The HLQ for general health literacy has a similar structure and even distinguishes nine scales (31). Moderate to large correlations with HLQ scales indicated good convergent validity. Similar to the HLQ and the HLS-EU (11), we also used a Likert scale for difficulty to represent the different competencies levels. The test information functions calculated by the GPCMs were peaked in the area below average for each factor, indicating that the subscales provide more information on the lower spectrum of PE-related health literacy. Consequently, the subscales may not be sound to differentiate between patients with high or very high PE-related health literacy. However, since distinguishing the levels in terms of limited health literacy seems more important to identify needs, we do not consider this aspect as a real limitation of our questionnaire.

Known-groups validity of the questionnaire was confirmed, as it was able to distinguish between different levels of education, job situations, and age groups. Our findings for “dealing with health information” are consistent with previous studies which revealed higher proportions of limited general health literacy in older patients and patients with low education and low social status (32, 33). Remarkably, the scores on the scale regarding “health-related selfcare” were higher in older patients, in unemployed patients and patients who were living alone. This may be explained by the fact that the time available for selfcare activities may be higher without a job or other family responsibilities that younger patients often face.

The investigation of the questionnaire’s responsiveness after receiving PE-related health information revealed small to large effects. Considering the fact that the brochure was a rather mild intervention and it could not be guaranteed whether the entire brochure has been read or how often the brochure has been used, the results are particularly good. Consequently, the questionnaire seems to be an appropriate tool to assess effects of intervention studies on PE-related health literacy.

We had only few missing values in our surveys and we excluded them for the validation process after inspection of their frequencies. For future studies, we recommend that one missing item per scale may be imputed, e.g., by the mean of the other items, otherwise the scale should not be calculated.

A strength of our study is the mixed-methods design with comprehensive involvement of patients who were directly affected by PE and could contribute by sharing their personal experiences. Since patients differ in age, history of cancer or other diseases, presence of symptoms, and medication intake, we tried to cover a broad spectrum of patients with PE in the interviews. The sample of the online survey was large enough to divide it into a test and validation sample. Our final questionnaire covers four domains, but with 23 self-administered items it is still short enough to be applied in a clinical setting. However, the study has some limitations. The sample of the evaluation study included many patients who were already part of a larger PE study cohort. Therefore, participants may be more sensitive toward study participation, have less long-term consequences after PE and may not represent the whole range of patients with PE. Cognitive debriefing was only conducted once for each item due to the large number of items included in the first draft of the item pool. The ability to detect change was also only tested for a small part of the sample in the evaluation study. Due to the postal survey, we were dependent on when patients returned their questionnaires to us and the time gap between the first and the second assessment were not the same for all participants. We were able to investigate many indicators of psychometric quality but we did not examine test–retest reliability to test the stability of the measure, which should be part of future studies.

## 5. Conclusion

The newly developed German HeLP questionnaire comprises 23 items in four domains and is the first instrument to measure health literacy in patients with PE. Overall, the questionnaire shows good validity and reliability. Further evaluation of the applicability of the questionnaire in different samples with patients with PE is required. The questionnaire can be used to identify patients with low health literacy who may require additional support from the healthcare system and to evaluate the impact of disease-specific interventions to improve PE-related health literacy.

## Data availability statement

The datasets presented in this article are not readily available because of ethical restrictions. The study participants did not agree that their data will be used by other researchers. Requests to access the datasets should be directed to SF, simone.fischer@med.uni-augsburg.de.

## Ethics statement

The studies involving humans were approved by Ethics Committee of the Ludwig-Maximilians-Universität München, Pettenkoferstr. 8a, 80336 Munich (Date of approval: 6 July 2020. Reference number: 20–452). The studies were conducted in accordance with the local legislation and institutional requirements. The participants provided their written informed consent to participate in this study.

## Author contributions

SF organized and conducted the interviews, performed the statistical analyses, and drafted the first version of the manuscript. CK and AA developed the interview guide, conducted the interviews, and helped to revise the manuscript. AK conducted the interviews, was involved in designing and supervising the study, and provided critical feedback on the manuscript. DH-S organized the data collection of the online study and provided critical feedback as an advisor. TB was involved in the organization of the evaluation study and supported the interpretation of the results from a medical perspective. HB and CM were involved in planning and supervising the research and provided critical feedback on the manuscript. IK designed and supervised the study, supported the interpretation of the results, and participated in the writing and critical revision of the manuscript. The brochure with evidence-based information for patients with PE was developed by AA, CK, AK, and HB. All authors contributed to the article and approved the submitted version.

## Funding

The study was funded by the Federal Joint Committee (G-BA) Germany (grant number: 01VSF19023).

## Conflict of interest

The authors declare that the research was conducted in the absence of any commercial or financial relationships that could be construed as a potential conflict of interest.

## Publisher’s note

All claims expressed in this article are solely those of the authors and do not necessarily represent those of their affiliated organizations, or those of the publisher, the editors and the reviewers. Any product that may be evaluated in this article, or claim that may be made by its manufacturer, is not guaranteed or endorsed by the publisher.
