# Supplementary material for: Health literacy in patients with pulmonary embolism: development and validation of the HeLP (Health Literacy in Pulmonary Embolism)-Questionnaire
Source: Front Public Health. 2023 Aug 29;11:1167499. doi: 10.3389/fpubh.2023.1167499 (PMC10497959; doi:10.3389/fpubh.2023.1167499)
Supplement: Supplementary file 1 [file Data_Sheet_1.PDF]

Supplementary Material

Health literacy in patients with pulmonary embolism: development and validation of the HeLP (Health Literacy in Pulmonary Embolism)-Questionnaire

Simone Fischer*, Anja Kalch, Constanze Küchler, Aliscia Rebecca Albani, Helena Bilandzic, Dirk Horenkamp-Sonntag, Thomas M. Berghaus, Christine Meisinger, Inge Kirchberger

*** Correspondence:** Simone Fischer: simone.fischer@med.uni-augsburg.de

Table S1: Sample characteristics of interviewees

Nr.	Age	Sex	Number of PE events	Time between interview and last PE event	Family status	School education	Ongoing anticoagulative medication
**1st Round: Concept of PE-sepcific health literacy (n=15)**							
**1**	56	m	1	2 years, 9 months	Divorced	≥ 12 years	Yes
**2**	56	m	1	7 years, 3 months	Married	≥ 12 years	Yes
**3**	50	m	2	5 years, 3 months	Single	≥ 12 years	Yes
**4**	79	m	1	1 month	Single	9 years	Yes
**5**	76	m	1	3 months	Married	9 years	Yes
**6**	34	m	1	6 months	Married	≥ 12 years	Yes
**7**	63	f	1	2 months	Married	10 years	Yes
**8**	53	m	1	10 months	Married	9 years	Yes
**9**	73	m	2	8 months	Divorced	10 years	Yes
**10**	46	f	1	2 years, 5 months	Married	≥ 12 years	Yes
**11**	72	f	2	8 years, 6 months	Married	9 years	Yes
**12**	19	f	1	1 year, 2 months	Single	≥ 12 years	No
**13**	33	m	3	6 years, 11 months	Single	≥ 12 years	No
**14**	49	f	1	10 months	Married	≥ 12 years	Yes
**15**	29	f	1	1 year, 4 months	Single	≥ 12 years	No
**2nd Round: Cognitive debriefing with first item pool (n=3)**							
**1**	49	f	1	1 year, 3 months	Married	≥ 12 years	Yes
**2**	34	m	1	1 year	Married	≥ 12 years	No
**3**	46	f	1	2 years, 11 months	Married	≥ 12 years	Yes
**3rd Round: Evaluating first version of the questionnaire (n=11)**							
**1**	56	m	2	1 year, 3months	Married	≥ 12 years	Yes
**2**	29	f	1	1 year, 10 months	Single	≥ 12 years	No
**3**	48	f	1	9 months	Married	9 years	Yes
**4**	22	f	1	2 years, 2 months	Single	≥ 12 years	Yes
**5**	57	m	1	8 years	Married	≥ 12 years	Yes
**6**	64	m	2	10 months	Married	9 years	Yes
**7**	46	f	1	3 years, 1 month	Married	≥ 12 years	Yes
**8**	49	f	1	1 year, 6 months	Married	≥ 12 years	Yes
**9**	34	m	1	1 year, 3 months	Married	≥ 12 years	No
**10**	20	f	1	1 year, 10 months	Single	≥ 12 years	No
**11**	71	m	1	1 year, 3months	Divorced	≥ 12 years	Yes

Table S2: Sample Characteristics of online survey

	All	Test sample	Validation sample
Variable	N = 1013	N= 505	N=508
**Age**			
18-30	9.0 (0.9)	5.0 (1.0)	4.0 (0.8)
31-40	21.0 (2.1)	9.0 (1.8)	12.0 (2.4)
41-50	53.0 (5.2)	23.0 (4.6)	30.0 (5.9)
51-60	192.0 (19.0)	93.0 (18.4)	99.0 (19.5)
61-70	294.0 (29.0)	161.0 (31.9)	133.0 (26.2)
71-80	266.0 (26.3)	137.0 (27.1)	129.0 (25.4)
> 80	178.0 (17.6)	77.0 (15.2)	101.0 (19.9)
**Sex**			
Male	664 (65.9)	334 (66.4)	330 (65.3)
Female	344 (34.1)	169 (33.6)	175 (34.7)
Missing	5	2	3
**Maritial status**			
Married	743 (73.4)	378 (75.0)	365 (71.9)
Unmarried	89 (8.8)	46 (9.1)	43 (8.5)
Divorced	100 (9.9)	43 (8.5)	57 (11.2)
Widowed	80 (7.9)	37 (7.3)	43 (8.5)
Missing	1	1	-
**Education**			
University degree	339 (33.7)	180 (36.0)	159 (31.4)
High school (≥ 12 years, „Abitur“)	202 (19.9)	100 (19.8)	102 (20.1)
Secondary school (10 years, “Mittlere Reife”)	236 (23.5)	122 (24.4)	114 (22.5)
Main school (9 years, “Hauptschulabschluss”)	225 (22.4)	97 (19.4)	128 (25.3)
No graduation	4 (0.4)	1 (0.2)	3 (0.6)
Missing	7	5	2
**Number of PE events**			
1	825 (81.4)	411 (81.4)	414 (81.5)
2	148 (14.6)	71 (14.1)	77 (15.2)
> 2	40 (3.9)	23 (4.6)	17 (3.3)
**Time since last PE event (months)**	18 (10, 29)	17 (10, 28)	19 (11, 29)
**Anticoagulant therapy**	984 (97.1)	491 (97.2)	493 (97.0)
**Prior diseases**			
Thrombophilia	219 (21.6)	106 (21.0)	113 (22.2)
Diabetes mellitus	163 (16.1)	75 (14.9)	88 (17.3)
Hypertension	566 (55.9)	280 (55.4)	286 (56.3)
Heart failure	190 (18.8)	77 (15.2)	113 (22.2)
Myocardial infarction	98 (9.7)	47 (9.3)	51 (10.0)
Stroke	59 (5.8)	29 (5.7)	30 (5.9)
Psychiatric disease	81 (8.0)	36 (7.1)	45 (8.9)
Pulmonary hypertension	35 (3.5)	19 (3.8)	16 (3.1)
Cancer	193 (19.1)	98 (19.4)	95 (18.7)

n (%); Median (Q_{25}, Q_{75})

Table S3: Slopes and thresholds of GPCMs for each item

	a	b_1	b_2	b_3	b_4
Dealing with health information					
Item 01	1.36	-2.19	-1.35	0.18	1.62
Item 02	2.22	-2.09	-1.78	-0.85	1.05
Item 03	2.69	-2.23	-1.77	-0.25	1.02
Item 04	2.35	-2.10	-1.73	-0.70	0.74
Item 05	1.17	-1.57	-1.37	0.44	1.80
Item 06	1.61	-2.24	-1.69	-0.04	1.57
Item 07	0.73	-2.77	-1.51	0.52	2.28
Disease management					
Item 08	1.45	-2.10	-1.86	-0.73	0.72
Item 09	1.36	-2.02	-1.99	-1.93	-0.66
Item 10	1.46	-1.53	-2.26	-1.51	0.29
Item 11	2.67	-2.22	-1.64	-0.50	1.07
Item 12	2.19	-2.74	-2.05	-1.02	0.51
Health-related selfcare					
Item 13	1.85	-2.91	-1.58	-0.28	1.31
Item 14	3.23	-2.88	-1.80	-0.41	0.93
Item 15	3.72	-1.90	-1.45	-0.60	0.75
Item 16	4.00	-2.14	-1.42	-0.22	1.04
Item 17	1.76	-2.24	-1.56	-0.59	1.23
Item 18	1.30	-2.50	-1.37	-0.06	1.71
Social support					
Item 19	1.43	-1.72	-1.05	0.08	1.30
Item 20	1.19	-1.81	-0.69	-0.10	1.16
Item 21	1.33	-1.89	-2.06	-0.82	0.78
Item 22	1.29	-1.56	-0.82	0.11	1.43
Item 23	2.99	-1.53	-0.86	0.16	1.13

a: item discrimination parameter; b_j = response category difficulty parameter; GPCM: graded partial credit model

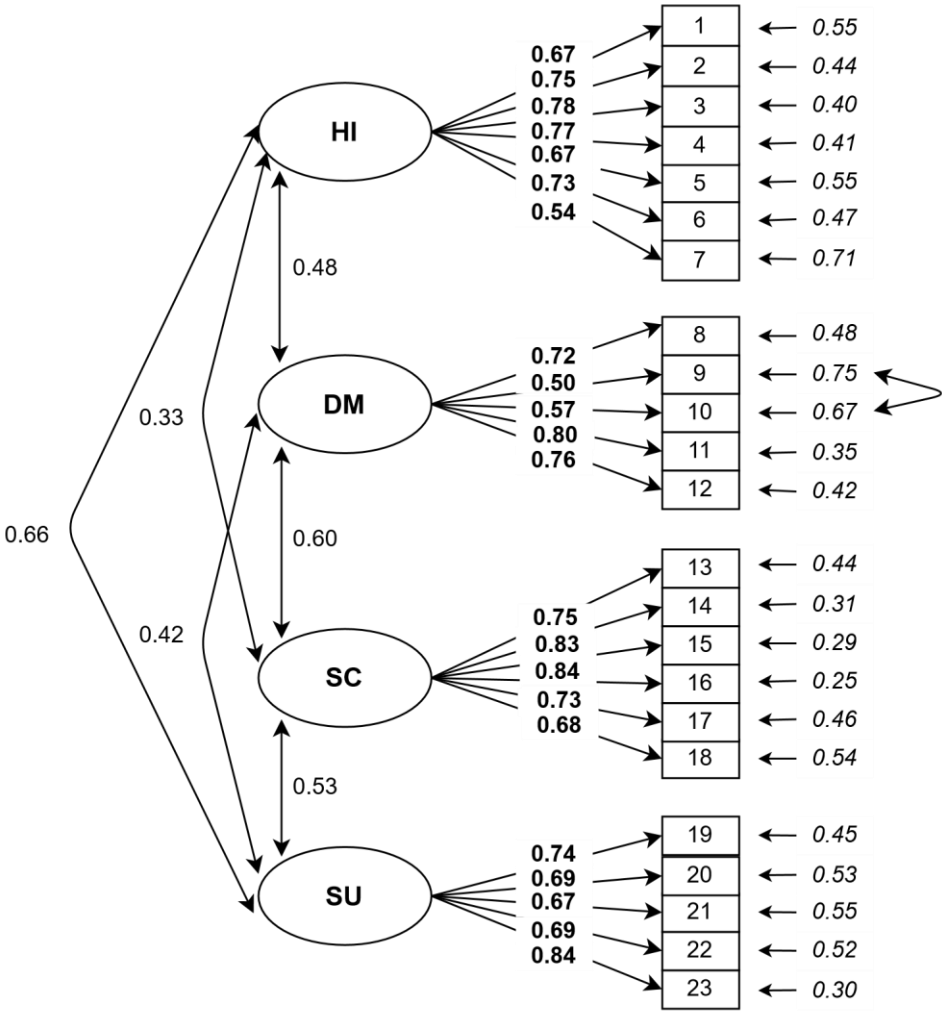

Figure S1: Standardized solution of the final CFA model. HI: Dealing with PE-related health information, DM: Disease management, SC: Health-related selfcare, SU: Social support. Factor loadings are in bold type, error variances are in italics, curved arrows represent co-varied error terms. All factor loadings were statistically significant with $p < 0.001$
